# Cytomixis doesn’t induce obvious changes in chromatin modifications and programmed cell death in tobacco male meiocytes

**DOI:** 10.3389/fpls.2015.00846

**Published:** 2015-10-12

**Authors:** Sergey Mursalimov, Natalya Permyakova, Elena Deineko, Andreas Houben, Dmitri Demidov

**Affiliations:** ^1^Institute of Cytology and Genetics, Siberian Branch, Russian Academy of SciencesNovosibirsk, Russia; ^2^Leibniz Institute of Plant Genetics and Crop Plant ResearchStadt Seeland, Germany

**Keywords:** Cytomixis, histone modifications, euchromatin/heterochromatin, synaptonemal complex, TUNEL assay, *Nicotiana tabacum*

## Abstract

Cytomixis is a poorly studied process of nuclear migration between plant cells. It is so far unknown what drives cytomixis and what is the functional state of the chromatin migrating between cells. Using immunostaining, we have analyzed the distribution of posttranslational histone modifications (methylation, acetylation, and phosphorylation) that reflect the functional state of chromatin in the tobacco microsporocytes involved in cytomixis. We demonstrate that the chromatin in the cytomictic cells does not differ from the chromatin in intact microsporocytes according to all 14 analyzed histone modification types. We have also for the first time demonstrated that the migrating chromatin contains normal structures of the synaptonemal complex (SC) and lacks any signs of apoptosis. As has been shown, the chromatin migrating between cells in cytomixis is neither selectively heterochromatized nor degraded both before its migration to another cell and after it enters a recipient cell as micronuclei. We also showed that cytomictic chromatin contains marks typical for transcriptionally active chromatin as well as heterochromatin. Moreover, marks typical for chromosome condensation, SC formation and key proteins required for the formation of bivalents were also detected at migrated chromatin.

## Introduction

Cytomixis is an enigmatic migration of nuclei between plant cells through intercellular channels of a special type (cytomictic channels), which are considerably larger than plasmodesmata. This phenomenon has been observed in 100s of higher plant species, mostly during microsporogenesis (for details, see the reviews by Lone and Lone, 2013 and Mursalimov et al., 2013b).

So far, the mechanisms underlying formation of cytomictic channels and migration of nuclei through them between plant microsporocytes have been studied in some detail. As has been shown, cytomixis is most frequently observed during meiotic prophase I (Negron-Ortiz, 2007; Liu et al., 2012; Barton et al., 2014). Cytomictic channels may be formed in the cell wall based on plasmodesmata or independently of them (Wang et al., 2002, 2004; Yu et al., 2004; Mursalimov et al., 2010, 2013a). Not just chromatin or chromosomes alone migrate through the cytomictic channels, rather the nucleus with all its components (chromatin, nuclear matrix, and nucleolus) enclosed within the intact nuclear membrane (Mursalimov and Deineko, 2011) can be transfered. In some cases, the nucleus may pass from a donor to a recipient cell as a whole to form binucleated microsporocytes (Mursalimov and Deineko, 2015); however, a more frequent situation is when the migrated fragments of the nucleus produce micronuclei in the cytoplasm of recipient cells (Mursalimov and Deineko, 2011, 2015; Barton et al., 2014).

It has been shown earlier that cytomixis may result in unreduced, polyploid, aneuploid, and sterile pollen (Falistocco et al., 1995; Ghaffari, 2006; Negron-Ortiz, 2007; Lavia et al., 2011; Pécrix et al., 2011; Mursalimov and Deineko, 2015). However, it is unclear what kind of chromatin migrates between cytomictic cells at the early meiotic stages, and what is the level of transcriptional activity and whether it has been damaged. To clarify the functional state of the migrating chromatin we analyzed the distribution of some histone modifications in cytomictic cells. The corresponding marks are highly conserved for all the eukaryotes and make it possible to detect euchromatic and heterochromatic regions, double-strand DNA breaks and the process of chromosome condensation and cohesion (Manzanero et al., 2000; Fuchs and Schubert, 2012; He et al., 2014).

In this work, we used immunostaining to analyze the distribution of major posttranslational histone modifications (methylation, acetylation, and phosphorylation) in tobacco cytomictic microsporocyte nuclei as well as in the micronuclei formed by cytomixis. Immunostaining and electron microscopy have allowed us to detect and examine the synaptonemal complex (SC) elements in the migrating chromatin and the cells involved in cytomixis have been analyzed for signs of DNA damage using internucleosomal DNA fragmentation and TUNEL assays.

## Materials and Methods

### Plant Material

*Nicotiana tabacum* L. cv. (2*n* = 4*x* = 48) Petit Havana SR1 line was used in the work. The plants were grown in a greenhouse with a photoperiod of 16/8 h (day/night) at a temperature of 22/18°C (day/night).

### Immunostaining of Squash Preparations

Anthers were fixed with freshly prepared 4% paraformaldehyde in Phosphate-buffered saline (PBS, pH 7.3) for 15–30 min on ice. Anthers were then washed in PBS 3 min × 15 min on ice, squashed into suspension in PBS and then microsporocyte cell walls were digested at 37°C for 30 min in a mixture of 1% Pectolyase (Sigma), 1% Cytohelicase (Sigma) 0.7% Cellulase R-10 (Duchefa), and 0.7% Cellulase (Calbiochem) dissolved in PBS. Cells were centrifuged at 7000 *g* for 6 min at 4°C, resuspended with PBS and centrifuged again at 7000 *g* for 6 min at 4°C. Then cells with a minimum volume of PBS (10–20 μl) were transferred to poly-L-lysine-slides and squashed between a glass slide and cover slip. After freezing in liquid nitrogen for 60 s, the cover slips were removed and the slides were transferred immediately into PBS.

To avoid non-specific antibody binding, slides were incubated for 1 h in PBS with 3% BSA and 0.1% Tween 20 at 37°C in a prewarmed moisture chamber, then primary antibodies were applied to the slides.

The primary antibodies used were mouse anti-histone H3S10ph (Abcam, cat. no. 14955); rabbit anti-H3K4me3 (Millipore, cat. no. 07-473); anti-H3K9me2 (Active Motif, cat. no. 39239); anti-H3K27me2 (EMD Millipore, cat. no. 07-452); anti-H3K4me2 (EMD Millipore, cat. no. ABE250); anti-H3T11ph (Upstate, cat. no. 07-492); anti-H2A.XS139ph (Cell Signaling, cat. no. 2577); anti-H3K9ac (Thermo Scientific, cat. no. MA5-11195); anti-H3K27me3 (EMD Millipore, cat. no. 07-449); anti-H3K27ac (Abcam, cat. no. 45173); anti-H3K14ac (Abcam, cat. no. 52946); anti-H3K18ac (Abcam, cat. no. 1191); anti-H2AT121ph (MyBioSource, cat. no. 004447); rat anti-H3Ser28ph (Sigma, cat. no. H9908); rabbit anti-Asy1 and guinea pig anti-Zyp1 from maize (kindly provided by Rachel Wang, Cande group, Berkeley). All the primary antibodies were diluted at 1:200 in PBS, 1% BSA, 0.1% Tween 20.

After overnight incubation at 4°C in a moisture chamber and washing three times for 15 min in PBS with 0.1% Tween 20, the slides were incubated for 1 h at 37°C in a prewarmed moisture chamber with Alexa Fluor 594- and DyLight 488-conjugated anti-rabbit IgG (Jackson ImmunoResearch, cat. no. 711-585-152 and 111-485-144); Alexa Fluor 488-conjugated anti-mouse IgG (Molecular probe, cat. no. A-11001); Alexa Fluor 594-conjugated anti-guinea pig IgG (Molecular probe, cat. no. A-11076); FITC-conjugated anti-rat IgG (Dianova, cat. No 112-095-003), diluted at 1:250 in PBS, 1% BSA, 0.1% Tween 20. Then the slides were washed three times for 15 min in PBS with 0.1% Tween 20, and mounted in ProLong Gold antifade (Life Technologies) containing 4′,6-diamidino-2-phenylindole (DAPI).

Fluorescent signals were observed using an epifluorescence microscope Olympus BX-61. Images were captured with an ORCA-ER CCD camera (Hamamatsu), analyzed, and processed with Adobe Photoshop CS4 software (Adobe Systems Incorporated). Single DAPI images were presented in gray as the best way to discriminate chromosome structures. In the merged images the DAPI staining is blue. From 100 to 200 nuclei were used for the immunostaining with each antibody.

### Immunostaining of PEG-Embedded Sections

Anthers at different stages of development were fixed, embedded in polyethylene glycol 1500, cut in semithin sections with a thickness of 10 μm on a LEICA RM2265 microtome (LEICA, Wetzlar, Germany) and immunostained according to Houben et al. (2011). Mouse anti-H3S10p (Abcam, cat. no. 14955) was used as a primary antibody and Alexa Fluor 488-conjugated anti-mouse IgG (Molecular probe, cat. no. A-11001) as a secondary one. The callose wall was stained with aniline blue (0.5% in PBS). Note that tobacco cells after PEG-embedding have cytoplasmic autofluorescence (presented in green with callose wall). Image capture and processing were performed as described above.

### Terminal Deoxynucleotidyl Transferase dUTP Nick End Labeling (TUNEL) Assay

Tobacco anthers were used like for immunostaining of chromosomes by squashing method. TUNEL assay was performed using DeadEnd Fluorometric TUNEL system (Promega, cat. no. G3250) according to the manufacturer’s instruction. Meiotic and somatic cells after heat shock were used as positive controls according to (Fan and Xing, 2004). Tobacco anthers and leaves that had been frozen in liquid nitrogen were sealed in plastic bags to prevent water access and heated in a water bath at 42°C for 120 min.

### Isolation of Nuclear DNA and DNA Laddering Analysis

Total DNA was isolated from anthers and leaves with CTAB (Sambrook et al., 1989). Then DNA was separated by electrophoresis in a 2% agarose gel. The gel was then stained with ethidium bromide for visualization. For positive control, somatic cells after heat shock were used (Fan and Xing, 2004).

### Electron Microscopy

Sample preparation for transmission electron microscopy was as described previously (Mursalimov and Deineko, 2012). Probes were studied in Libra120 (Carl Zeiss, Germany) transmission electron microscope at an accelerating voltage of 80 kV.

## Results

In order to assess the functional state of the chromatin migrating between cytomictic cells, we used immunostaining to analyze the distribution of main posttranslational histone modifications in intact tobacco microsporocyte nuclei, nuclei in the process of migration, and micronuclei formed in recipient cells as a result of cytomixis. In tobacco microsporogenesis, cytomixis is most frequently observed during meiotic prophase I (Sidorchuk et al., 2007; Mursalimov and Deineko, 2012, 2015). Correspondingly, we have analyzed the histone modifications in microsporocyte nuclei starting from the leptotene to metaphase I, after which the cytomictic chromatin is indistinguishable in dividing cells. In order to demonstrate migration of chromatin between cytomictic cells, cytoplasm autofluorescence signals, denoting cell boundaries were added to merged images.

### Migrating Chromatin is Transcriptionally Potent

The following types of histone modifications were selected as markers for transcriptionally potent chromatin: histone H3K4me2, H3K4me3, H3K9ac, H3K14ac, H3K18ac, and H3K27ac (Horst et al., 2009; Jang et al., 2011; Du et al., 2013).

**FIGURE 1:**
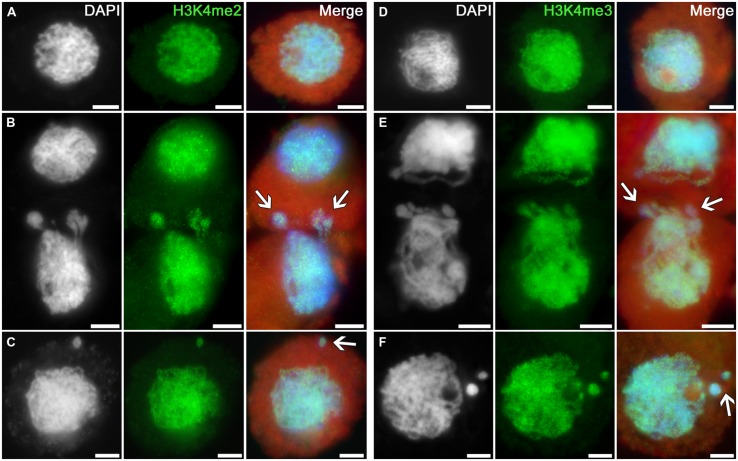
**Distributions of dimethylated and trimethylated histone H3K4 in the tobacco microsporocytes in meiotic prophase I by cytomixis. (A)** H3K4me2 and **(D)** H3K4me3 in intact microsporocytes; **(B)** H3K4me2 and **(E)** H3K4me3 in cytomictic microsporocytes (*arrows* denote the migrating part of nucleus that entered the recipient cell); and **(C)** H3K4me2 and **(F)** H3K4me3 in microsporocytes after cytomixis (*arrows* denote micronuclei). Red, cytoplasm autofluorescence in merged images; bars, 5 μm.

Intensive and uniform immunosignals were obtained in nuclei of intact tobacco microsporocytes in the zygotene–pachytene stage using antibodies recognizing dimethylated and trimethylated histone H3 at lysine 4 (**Figures 1A,D**). The signals are similarly distributed in cytomictic nuclei (**Figures 1B,E**; *arrows*). The intensity and distribution of immunopatterns in nuclei migrating between cells (**Figures 1B,E**; *arrows*) are identical to those in intact cell nuclei (**Figures 1A,D**). The signal in recipient cells, which thus have additional chromatin, also does not differ from the norm (**Figures 1B,E**). No differences are observed between the part of migrating nuclei still remaining in the donor cell versus the part that already entered the recipient cell (**Figures 1B,E**; *arrows*). The level of histone H3 di- and trimethylation at lysine 4 also remains unchanged in the micronuclei formed in recipient cells after cytomixis (**Figures 1C,F**; *arrows*).

**FIGURE 2:**
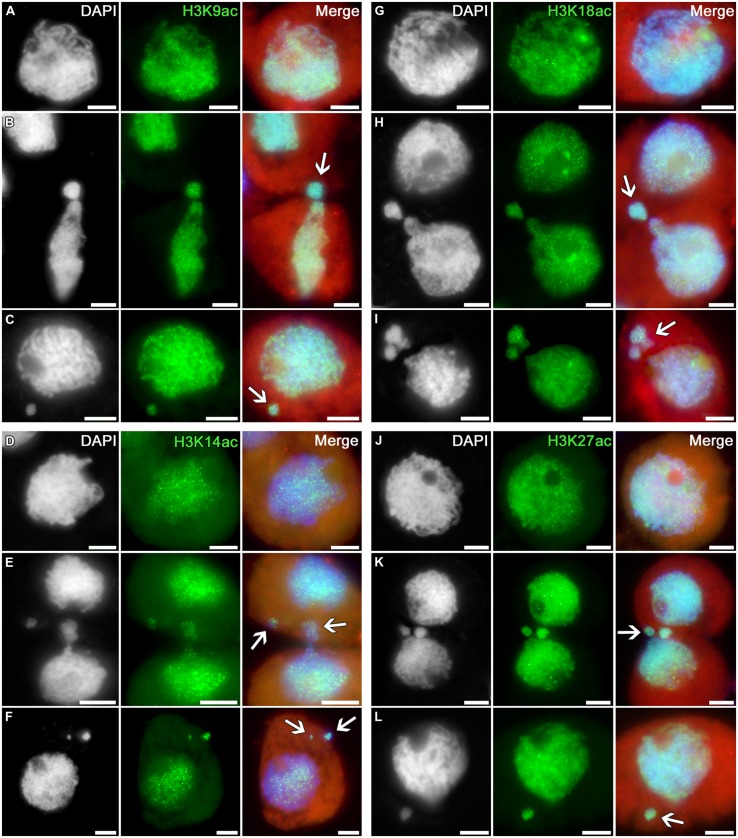
**Distributions of acetylated histone H3 in tobacco microsporocytes in meiotic prophase I by cytomixis. (A)** H3K9ac, **(D)** H3K14ac, **(G)** H3K18ac, and **(J)** H3K27ac in intact microsporocytes; **(B)** H3K9ac, **(E)** H3K14ac, **(H)** H3K18ac, and **(K)** H3K27ac in cytomictic microsporocytes (*arrows* denote the migrating part of nucleus that entered the recipient cell); **(C)** H3K9ac, **(F)** H3K14ac, **(I)** H3K18ac, and **(L)** H3K27ac in microsporocytes after cytomixis (*arrows* denote micronuclei). Red, cytoplasm autofluorescence in merged images; bars, 5 μm.

The profile of histone H3 acetylation at lysines 9, 14, 18, and 27 in tobacco microsporocytes is analogous to the H3K4 methylation profile (**Figure 2**). Intensive signals are uniformly distributed over the nuclei of intact cells (**Figures 2A,D,G,J**) as well as over the nuclei of cytomictic cells (**Figures 2B,E,H,K**). Chromatin in the process of migration to another cell (**Figures 2B,E,H,K**; *arrows*) does not display obvious differences in the distribution and intensity of the immunosignal as compared to the remaining part of the migrating nucleus still remaining in the donor cell. The micronuclei formed in recipient cells do not lose their normal acetylation level on completion of cytomixis (**Figures 2C,F,I,L**; *arrows*).

Thus, the histone H3 methylation and acetylation profiles in the chromatin migrating between cytomictic cells correspond to the profile of transcriptionally potent chromatin, and these markers do not disappear in the micronuclei formed in recipient cells after completion of cytomixis.

### The Heterochromatization Level of Migrating Chromatin does not Change

**FIGURE 3:**
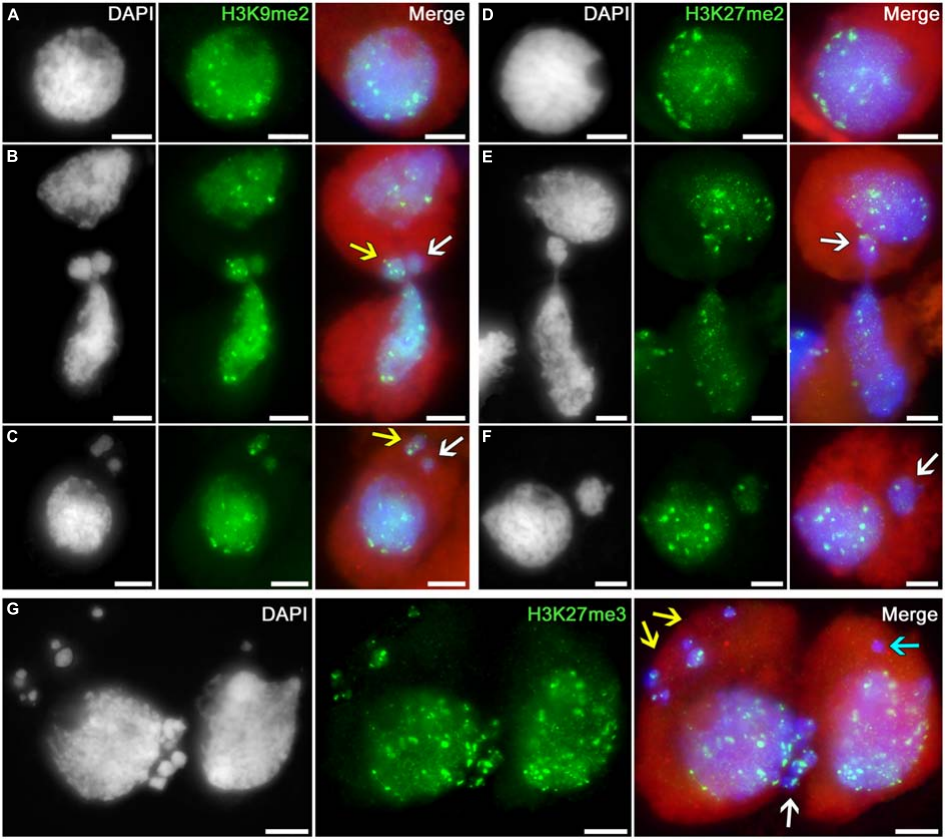
**Distributions of dimethylated histone H3K9 and di- and trimethylated H3K27 in tobacco microsporocytes in meiotic prophase I by cytomixis. (A)** H3K9me2 and **(D)** H3K27me2 in intact microsporocytes; **(B)** H3K9me2 and **(E)** H3K27me2 in cytomictic microsporocytes (*arrows* denote the migrating part of nucleus that entered the recipient cell); **(C)** H3K9me2 and **(F)** H3K27me2 in microsporocytes after cytomixis (*arrows* denote micronuclei); and **(G)** H3K27me3 in cytomictic microsporocytes (*white arrow*, the migrating part of nucleus that entered the recipient cell; *yellow* and *turquoise arrows*, micronuclei). Red, cytoplasm autofluorescence in merged images; bars, 5 μm.

The following histone modification types were selected as the markers for heterochromatin: H3K9me2, H3K27me2, and H3K27me3. Uniform immunosignals of medium intensity over the entire nuclei in tobacco microsporocytes were observed, in addition to individual bright signals, which most likely correspond to regions enriched in heterochromatin (**Figures 3A,D**).

The chromatin that migrates between cytomictic cells (**Figures 3B,E,G**; *arrows*) neither before nor after migration reveals any sign of additional heterochromatization. Moreover, the micronuclei formed after cytomixis are not hypermethylated at lysines 9 and 27 of histone H3 in the recipient cell (**Figures 3C,F,G**; *arrows*). Most likely, heterochromatic blocks are randomly distributed in the migrating nuclei. Several bright immunosignals are either observable in the migrating chromatin (**Figure 3B**, *yellow arrow;* **Figures 3E,G**; *white arrows*) or may be completely absent (**Figure 3B**, *white arrow*). Correspondingly, heterochromatin blocks may be present in different quantities in the micronuclei formed after cytomixis (**Figure 3C** *yellow arrow;* **Figure 3F**, *white arrow;* and **Figure 3G**, *yellow arrows*) or may be completely absent (**Figure 3C** *white arrow;* **Figure 3G**, *turquoise arrow*).

Thus, chromatin that leaves the cytomictic cell is neither previously heterochromatized (hypermethylated at lysines 9 and 27 of histone H3) nor inactivated during migration and upon entering the recipient cells as micronuclei.

### Migrated Chromatin Contains Marks of Histone Phosphorylation that Correlate with Condensation and Separation of Chromosomes

Phosphorylation of histones is an important marker of chromosome condensation and segregation during cell division, repair of damaged DNA, and apoptosis (Thiriet and Hayes, 2005; Houben et al., 2007). In cytomictic cells, we studied the distributions of the following histone phosphorylation types: H3S10ph, H3S28ph, H3T11ph, H2AT121ph, and H2A.XS139ph.

**FIGURE 4:**
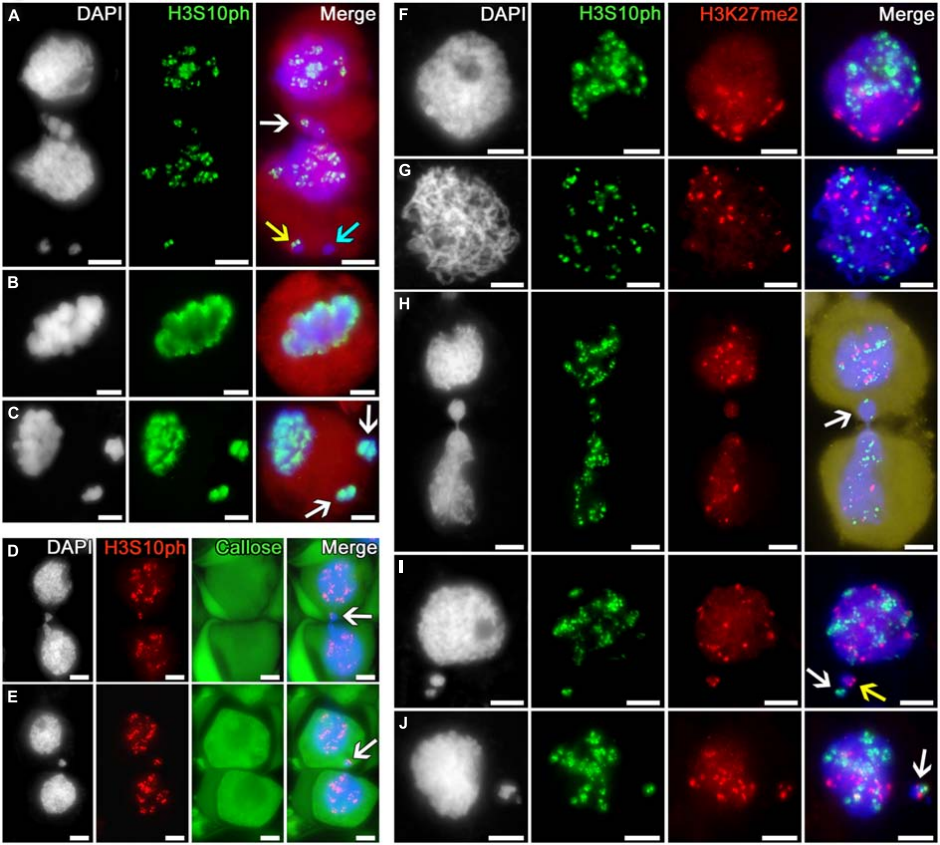
**Distribution of phosphorylated histone H3S10 in tobacco microsporocytes and its colocalization with dimethylated H3K27 by cytomixis. (A)** H3S10ph in cytomictic microsporocytes (*white arrow* denotes the migrating part of nucleus that entered the recipient cell; *yellow* and *turquoise arrows*, micronuclei); H3S10ph in **(B)** an intact microsporocyte and **(C)** a microsporocyte after cytomixis in meiotic metaphase I (*arrow*s, the chromatin of micronuclei; *red*, cell cytoplasm); **(D,E)** H3S10ph in cytomictic microsporocytes in sections of anthers embedded in PEG (*arrow*s, the migrating part of nucleus that entered the recipient cell). The callose wall was stained with aniline blue (cytoplasmic autofluorescence presented in green); **(F)** double labeling of H3S10ph and H3K27me2 in intact microsporocytes in the zygotene and **(G)** pachytene; **(H)** double labeling of H3S10ph and H3K27me2 in cytomictic microsporocytes (*arrow* denotes the migrating part of nucleus that entered the recipient cell; *yellow*, cell cytoplasm); and **(I,J)** double labeling of H3S10ph and H3K27me2 in the microsporocytes after cytomixis (*arrows* denote micronuclei); bars, 5 μm.

The phosphorylation of histone H3 at serine 10 in tobacco microsporocytes is initiated in early meiotic prophase I, being detectable as intensive double signals putatively localized to the pericentromeric regions of chromosomes (**Figures 4A,D–J**). In cytomixis, these signals are distributed over the migrating nuclei in a random manner (**Figure 4A**): they are detectable in the migrating part of the nucleus (**Figure 4A**, *white arrow*) and appear in micronuclei (**Figure 4A**, *yellow arrow*) or may be absent in the migrating chromatin, as evidenced by micronuclei lacking the signal (**Figure 4A**, *turquoise arrow*).

By meiotic metaphase I, the signal indicating the phosphorylation of histone H3 at serine 10 spreads along the entire chromosome lengths (**Figure 4B**). At the same stage, the chromatin of both the recipient cell and cytomictic micronuclei after cytomixis undergo normal phosphorylation as in the intact nuclei (**Figure 4C**).

Thus, microsporocytes display normal phosphorylation of histone H3 at serine 10 dynamics in both the chromatin migrating between cells in cytomixis and the cytomictic micronuclei, matching the current meiotic stage and the degree of chromosome condensation.

Antibodies against histone H3S10ph modification was selected for comparing the cytomixis patterns in anther squash preparations and sections of the anthers embedded in PEG with retained native cell structure (**Figures 4D,E**). Examination of these sections demonstrates that the cytomixis pattern in the specimens embedded in PEG with preserved cell wall does not differ from that observed in squash preparations. Nuclei similarly migrate between cells in the zygotene–pachytene and form micronuclei displaying different quantities of immunosignals in recipient cells (**Figures 4D,E**, *arrows*).

Thus, we have demonstrated that the manipulations used for squash preparations do not influence the cytological pattern of cytomixis in tobacco microsporocytes, thereby proving that the immunostaining results thus obtained are adequate.

Double immunostaining of tobacco microsporocyte nuclei in the zygotene (bouquet stage) with anti-H3S10ph and H3K27me2 showed a clustering of immunosignals at the opposite poles of the nuclei (**Figure 4F**). At pachytene, this clustering disappears, and the signals spread over the nucleus but do not colocalize (**Figure 4G**). In case of cytomixis, the H3S10ph and H3K27me2 signals independently appear in the migrating part of the nucleus (**Figure 4H**, *arrow*). Cytomictic micronuclei may contain only H3S10ph signal (**Figure 4I**, *white arrow*), only H3K27me2 signal (**Figure 4I**, *yellow arrow*), or both signals at once (**Figure 4J**, *arrow*).

**FIGURE 5:**
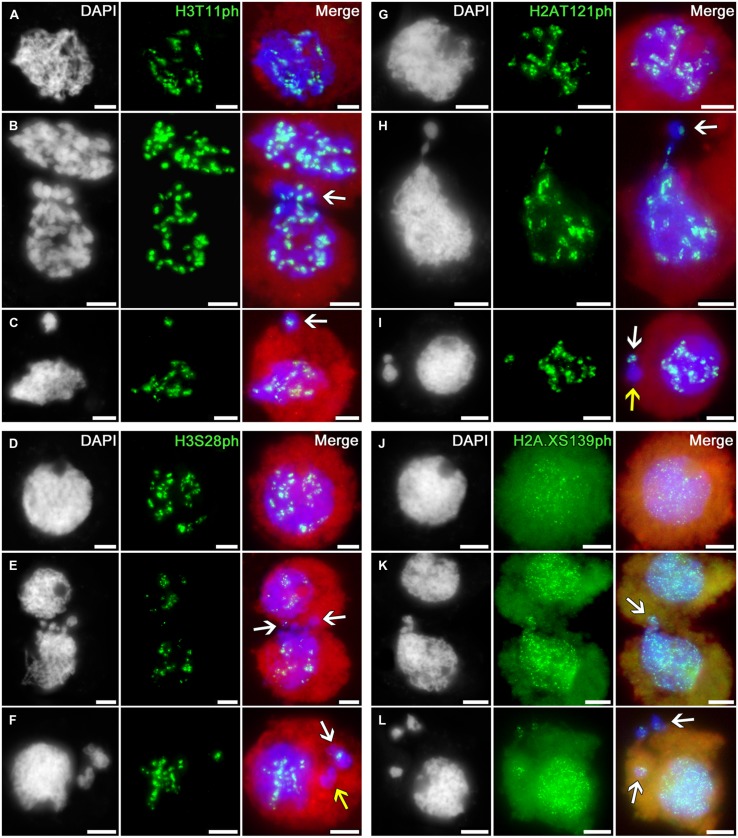
**Distributions of phosphorylated histones H3T11, H3S28, H2AT121, and H2A.XS139 in tobacco microsporocytes in meiotic prophase I by cytomixis. (A)** H3T11ph, **(D)** H3S28ph, **(G)** H2AT121ph, and **(J)** H2A.XS139ph in intact microsporocytes; **(B)** H3T11ph, **(E)** H3S28ph, **(H)** H2AT121ph, and **(K)** H2A.XS139ph in cytomictic microsporocytes (*arrows* denote the migrating part of nucleus that entered the recipient cell); and **(C)** H3T11ph, **(F)** H3S28ph, **(I)** H2AT121ph, and **(L)** H2A.XS139ph in microsporocytes after cytomixis (*arrows* denote micronuclei). Red, cytoplasm autofluorescence in merged images; bars, 5 μm.

The immunosignals indicating phosphorylation of histone H3 at threonine 11 (a marker of chromatin condensation in cell division (Houben et al., 2005)) and serine 28 (coupled with sister chromatid cohesion in cell division (Gernand et al., 2003)) and phosphorylation of histone H2A at threonine 121 (involved in Shugoshin binding, preserving cohesion of centromeric regions in cell division; Wang et al., 2012) during meiotic prophase I display the distributions similar to H3S10ph in intact tobacco microsporocyte nuclei, migrating nuclei in cytomixis, and the micronuclei formed after cytomixis (**Figures 5A–I**).

The migrating chromatin may contain different numbers of immunosignals (**Figures 5B,E,H**; *arrows*) whereas the micronuclei formed after cytomixis may either contain the signals (**Figures 5C,F,I**; *white arrows*) or lack them (**Figures 5F,I**; *yellow arrows*).

Thus, the phosphorylation patterns of histones H3 and H2A in cytomictic nuclei and micronuclei do not differ at all from the phosphorylation of these histones in intact microsporocyte nuclei, and match the normal degree of phosphorylation characteristic of the current meiotic stage.

The phosphorylation of histone H2A.X at serine 139 (γH2AX) is a marker of DNA double-strand breaks (Rogakou et al., 1999; Xiao et al., 2009). In the zygotene–pachytene, anti-H2A.XS139ph detects in tobacco microsporocytes numerous small signals dispersed over both the intact (**Figure 5J**) and migrating (**Figure 5K**) nuclei. Emergence of such double-strand DNA breaks is associated with recombination processes, taking place at this meiotic stage (Chicheportiche et al., 2007). There are no visible changes in the number of immunosignals in migrating chromatin (**Figure 5K**, *arrow*) or in cytomictic micronuclei (**Figure 5L**, *arrows*).

### Migrating Chromatin Contains Synaptonemal Complex

The SC is a chromosome structure formed during meiotic prophase I that provides a physical connection between homologous chromosomes. We have examined the presence of SC structures in cytomictic chromatin using electron microscopy and immunostaining for the SC proteins ZIP1 and ASY1.

**FIGURE 6:**
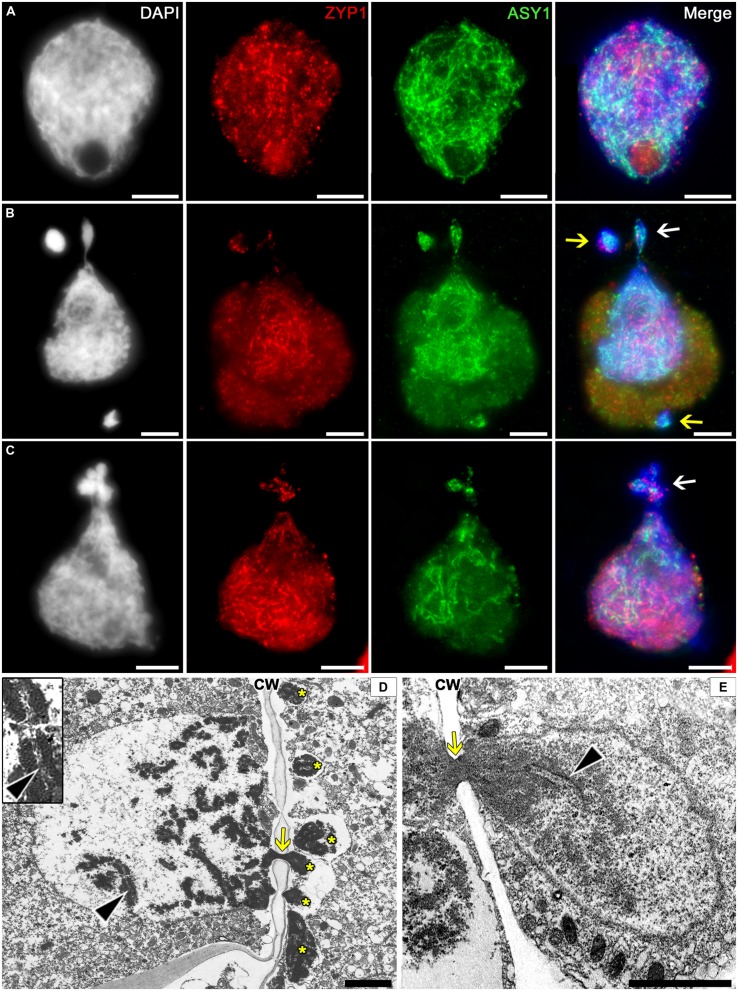
**Localization of the synaptonemal complex (SC) in tobacco cytomictic microsporocytes by **(A–C)** immunostaining and **(D,E)** electron microscopy.** Distributions of the ZIP1 and ASY1 proteins in **(A)** intact microsporocytes; **(B,C)** cytomictic microsporocytes (*white arrows* denote the migrating part of nucleus and *yellow arrows*, micronuclei); **(D,E)** the SC (*black arrowheads*) in migrating nuclei (*asterisks*, the part of the nucleus that entered recipient cell; *yellow arrows*, cytomictic channel filled with chromatin; and *CW*, cell wall); bars, 5 μm in **(A–C)** and 2 μm in **(D,E)**.

The immunostaining for SC proteins in tobacco microsporocytes has demonstrated that the nuclei in zygotene–pachytene contain the ZIP1 and ASY1 proteins, which do not colocalize with one another (**Figure 6A**). Most likely, the transverse filament proteins ZIP1 appear after the axial SC elements, ASY1, disappear. The cytomictic cells display the immunosignals of both proteins (**Figures 6B,C**), which do not colocalize and gradually come and go during prophase, similar to the pattern characteristic of intact cells. Depending on the prophase stage, either ASY1 (**Figure 6B**, *arrows*) or ZIP1 (**Figure 6C**, *arrow*) may be prevalent in the migrating chromatin.

In the electron micrographs, SC is visible in the migrating nucleus before chromatin enters the cytomictic channel (**Figure 6D**, *black arrowheads*), while the chromatin inside the channels becomes denser and looks as a black structure (**Figures 6D,E**, *yellow arrows*) with undetectable SC elements. However, the chromatin restores its structure immediately upon leaving the cytomictic channel and entering the recipient cell (**Figure 6E**, *black arrowhead*).

Thus, immunostaining and electron microscopy confirm the presence of SC-like structures in the migrating chromatin and the micronuclei formed in the recipient cells after cytomixis.

### The Cytomictic Cells Lack Any Signs of Apoptotic DNA Degradation

In order to test whether the processes of programmed cell death (PCD) are triggered in the cell after it has acquired “surplus” chromatin owing to cytomixis, we have searched for the signs of DNA damage characteristic of PCD. The signs suggesting internucleosomal DNA fragmentation were searched for by electrophoretic separation of the genomic DNA isolated from tobacco microsporocytes in the zygotene–pachytene (when the cytomictic rate is maximum). The TUNEL assay was used to search *in situ* for the signs of DNA damage in cytomictic tobacco microsporocytes.

**FIGURE 7:**
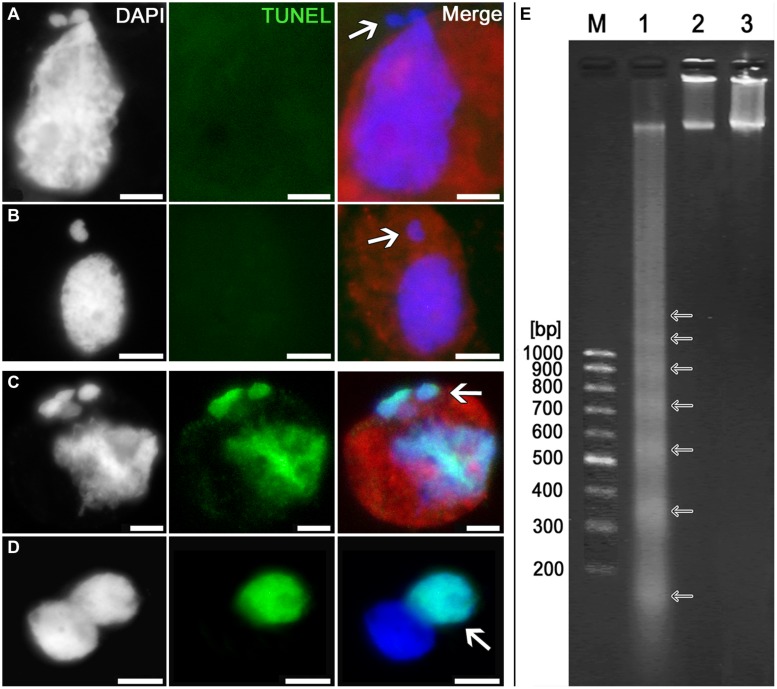
**Detection of DNA fragmentation in cytomictic cells by TUNEL assay and agarose gel electrophoresis.** TUNEL assay data for the tobacco microsporocytes **(A)** by cytomixis (*arrow* denotes the migrating part of the nucleus) and **(B)** after cytomixis (*arrow* denotes the micronucleus); positive control after heat shock of **(C)** microsporocytes after cytomixis (*arrows* denote micronuclei) and **(D)** somatic cells (*arrow*, TUNEL-positive nucleus); bars, 5 μm. **(E)** DNA electrophoretic pattern (agarose gel): M, molecular weight marker; 1, positive control; 2, DNA of untreated microsporocytes; and 3, DNA of untreated leaf cells.

TUNEL assay has shown that the cytomictic cells display no signs of DNA degradation both when the nuclei migrate through the cytomictic channel and on completion of migration and formation of micronuclei (**Figures 7A,B**; *arrows*). The nucleus of a recipient cell after the micronuclei are formed also displays no signs of DNA degradation (**Figure 7B**). TUNEL assay gave positive results only for the positive control, namely, the tobacco microsporocytes with cytomictic micronuclei (**Figure 7C**, *arrow*) and somatic anther cells (**Figure 7D**, *arrow*) heat-shocked to induce apoptosis-like changes. The test for the presence of internucleosomal DNA fragmentation was also positive only in the case of the positive (heat-shocked) control (**Figure 7E**, *lane 1*), while the genomic DNA isolated from intact tobacco microsporocytes (**Figure 7E**, *lane 2*) and leaf cells (**Figure 7E**, *lane 3*) displayed no signs of internucleosomal fragmentation. Since only up to 4% of cells exhibiting cytomixis in SR1 plants (Sidorchuk et al., 2007), a large amount (5 μg) of genomic DNA was assayed, but even in this case no DNA fragmentation was detected. Thus, any signs of the DNA damages characteristic of PCD are absent in the tobacco microsporocytes undergoing cytomixis.

## Discussion

Cytomixis was discovered over a century ago; however, the mechanism driving the migration of chromatin from one cell to another is still vague. On the one hand, it is believed that cytomixis is a pathological process associated with elimination of damaged chromatin/cells (Kravets, 2011; Barton et al., 2014). On the other hand, cytomixis is assumed to produce gametes with different chromosomes number (Falistocco et al., 1995; Ghaffari, 2006; Negron-Ortiz, 2007; Pécrix et al., 2011; Lavia et al., 2011). Yet none of these hypotheses have any direct experimental confirmation. First and foremost, this is associated with the absence of methods enabling the tracing of a microsporocyte from the moment when it acquired additional chromatin by cytomixis to the moment when it gives a tetrad of microspores and then gametes. Moreover, it is impossible to unambiguously identify migrated chromatin in microsporogenesis after meiotic metaphase I, and far less to prove its cytomictic origin, since no specific markers for cytomictic chromatin have been yet discovered.

Nonetheless, we have succeeded in demonstrating that the migrating chromatin in case of cytomixis in tobacco microsporogenesis lacks any signs of altered chromatin structure and that the cells involved in this process display no signs of PCD.

### Cytomixis does not Change the State of Migrating Chromatin

If either migrating chromatin or whole cells were degraded in case of cytomixis, the markers characteristic of this process would be detectable. One of the most illustrative examples of such chromatin degradation is the meiotic silencing and fragmentation of the male zebra finch germline-restricted chromosome (Goday and Pigozzi, 2010; Schoenmakers et al., 2010). In this case, the chromatin is eliminated from the nucleus during male meiotic prophase I and undergoes characteristic changes in its histone modifications. The eliminated chromatin loses the di- and trimethylation of histone H3 at lysine 4 (markers of euchromatin), is subject to pronounced di- and trimethylation of histone H3 at lysine 9 (markers of heterochromatin) as well as to surplus phosphorylation of histone H2A.X at serine 139 (marker of DNA double-strand breaks), thus becoming TUNEL-positive (Goday and Pigozzi, 2010; Schoenmakers et al., 2010). Such changes are characteristic of the inactivated chromatin destined to be eliminated. However, despite that cytomixis in tobacco takes place at the same stage of male meiosis as chromosome elimination in the zebra finch, the analogous changes in tobacco cytomictic chromatin are undetectable.

Using six euchromatin markers – H3K4me2, H3K4me3, H3K9ac, H3K14ac, H3K18ac, and H3K27ac—we have demonstrated that the chromatin migrating between cytomictic cells does not change its state. These chromatin marks also retained in the micronuclei formed after cytomixis in recipient cells. These data are also confirmed by analysis of the distributions of heterochromatin markers in migrating chromatin, namely, H3K9me2, H3K27me2, and H3K27me3. Before migration to another cell, the chromatin is not selectively heterochromatized nor is it inactivated in the micronuclei after cytomixis. The constitutive heterochromatin blocks detectable in tobacco microsporocyte nuclei are not associated with cytomixis and are randomly included into the migrating chromatin; note that there are no obvious changes of immunosignals in the micronuclei after cytomixis.

Analysis of the key markers for DNA damage—H2A.XS139ph (γH2AX), TUNEL, and DNA internucleosomal fragmentation—also suggests the absence of changes in migrating chromatin. When entering another cell and upon cytomixis completion, the migrating chromatin displays no signs of DNA damage. Any signs of DNA damage are also absent in the nuclei of recipient cells.

In the case of the zebra finch, the germline-restricted chromosome fragments remain detectable in the cell cytoplasm as a densely packed chromatin body until the end of meiosis (Goday and Pigozzi, 2010; Schoenmakers et al., 2010). Micronucleation is also observed in plant microsporogenesis, for example, of *Avena sativa* and *Glycine max*, when part of chromosomes remains beyond the nucleus during division, further separating as affected small microsporocytes, and eventually giving small sterile pollen (Baptista-Giacomelli et al., 2000; Ortega and Dorrance, 2011). Despite careful analysis, we have not detected any small microsporocytes during microsporogenesis of tobacco plants (Mursalimov and Deineko, 2015).

Thus, our data do not support the hypothesis that the migrated chromatin is degraded in case of cytomixis, or the cells involved in this process die before the tetrad is formed.

### Migrated Chromatin Contains Histone Marks Which Correlate with Chromosome Segregation

The phosphorylation of histone H3 at serines 10 and 28 and threonine 11, as well as of histone H2A at threonine 121, is critical for chromosome condensation and segregation in dividing cells (Manzanero et al., 2000; Houben et al., 2005, 2007; Kawashima et al., 2010).

We have shown that the phosphorylation of histones in the cytomictic microsporocytes does not differ from the normal process. The distribution of the above listed markers assumes correct chromatin condensation and segregation. Moreover, the case study of H3S10ph shows that the phosphorylation of histones also continues after migration in the micronuclei formed as a result of cytomixis. After completion of cytomixis, the micronuclei carrying the H3S10ph signal as individual loci are formed in tobacco microsporocytes in the pachytene; however, the chromatin in micronuclei by metaphase I is phosphorylated over the entire length, as during normal meiosis. This suggests that the recipient cell discerns no difference between the introduced and its own chromatin, so that the former is subject to further phosphorylation and receives the same stage-specific signals from the cytoplasm as the chromatin of the own recipient cell nuclei. Since the phosphorylation of histone H3 at serines 10, 28 as well as of histone H2A at threonine121 is coupled with active function of the centromeres and, this suggests a normal function of the latter in the migrating chromatin.

The presence of the phosphorylation signal of histone H2A.X at serine 139 in the cytomictic chromatin during zygotene–pachytene implies the presence of double-strand DNA breaks, which are associated with recombination, taking place at this meiotic stage (Chicheportiche et al., 2007). This suggests that recombination in the migrating chromatin is similar to the remaining chromatin of microsporocytes at this meiotic stage. However, recombination is feasible only provided that chromosomes are paired and bivalents are likely formed.

Electron microscopy examination and immunostaining of the SC proteins ZIP1 and ASY1 in tobacco microsporocytes confirm the presence of SC in the migrating chromatin. Note also that the ZIP1 and ASY1 proteins were only detected in microsporocyte nuclei in the zygotene–pachytene, and do not colocalize as in *Arabidopsis* (Higgins et al., 2005). Most likely, the filament proteins ZIP1 appear after the axial SC elements, ASY1, disappear, as is observed in the wheat (Khoo et al., 2012). Thus, the migrating chromatin shows histone marks for correct chromosome condensation and segregation according to the expected state at this meiotic stage, and furthermore contain key protein components of SC.

Signals of double labeling with antibodies against H3S10ph and H3K27me2 in tobacco microsporocytes has shown that they cluster at the opposite poles of the nucleus in the zygotene–pachytene (bouquet) stage, which is characteristic of centromeric and telomeric chromosome regions (Wen et al., 2012; Zhang et al., 2013). The H3S10ph signal is detected in the pericentromeric chromosome regions in mitosis and the second meiotic division (Manzanero et al., 2000; Houben et al., 2007); there are good grounds to suppose that phosphorylation of H3S10 in meiotic prophase I starts in the pericentromeric chromosome regions (Oliver et al., 2013). It is also known that the H3 dimethylated at lysine 27 can be detected in subtelomeric chromosome regions (Carchilan et al., 2007). The clustering of the H3K27me2 signal at the nuclear pole opposite to the centromeres at the bouquet stage suggests that this signal in tobacco microsporocyte nuclei marks the subtelomeric regions of some chromosomes. This assumption allows us to assess the integrity of the chromosomes migrating to other cells in case of cytomixis. In particular, the micronuclei that carry the “centromeric” signal and several “telomeric” ones are observed in the recipient cells after cytomixis, which is characteristic of a whole chromosome. The probability that the cytomictic chromatin containing centromeres and telomeres represents a normal bivalent remains to be tested. The fate of the cytomictic chromatin where only one of the signals is detected is questionable.

Thus, we have demonstrated that the chromatin migrating from one cytomictic cell to another shows no signs of chromatin alterations and DNA damage.

## Conflict of Interest Statement

The authors declare that the research was conducted in the absence of any commercial or financial relationships that could be construed as a potential conflict of interest.
